# Generation of Artificial Gamete and Embryo From Stem Cells in Reproductive Medicine

**DOI:** 10.3389/fbioe.2020.00781

**Published:** 2020-07-22

**Authors:** Pu-Yao Zhang, Yong Fan, Tao Tan, Yang Yu

**Affiliations:** ^1^Clinical Stem Cell Research Center, Peking University Third Hospital, Beijing, China; ^2^Beijing Key Laboratory of Reproductive Endocrinology and Assisted Reproductive Technology and Key Laboratory of Assisted Reproduction, Ministry of Education, Center for Reproductive Medicine, Department of Obstetrics and Gynecology, Peking University Third Hospital, Beijing, China; ^3^Key Laboratory for Major Obstetric Diseases of Guangdong Province, The Third Affiliated Hospital of Guangzhou Medical University, Guangzhou, China; ^4^Yunnan Key Laboratory of Primate Biomedical Research, Institute of Primate Translational Medicine, Kunming University of Science and Technology, Kunming, China

**Keywords:** artificial gametes, artificial embryogenies, stem cell, reproductive medicine, gametogenesis, embryo development

## Abstract

In addition to the great growing need for assisted reproduction technologies (ART), additional solutions for patients without functional gametes are strongly needed. Due to ethical restrictions, limited studies can be performed on human gametes and embryos; however, artificial gametes and embryos represent a new hope for clinical application and basic research in the field of reproductive medicine. Here, we provide a review of the research progress and possible application of artificial gametes and embryos from different species, including mice, monkeys and humans. Gametes specification from adult stem cells and embryonic stem cells (ESCs) as well as propagation of stem cells from the reproductive system and from organized embryos, which are similar to blastocysts, have been realized in some nonhuman mammals, but not all achievements can be replicated in humans. This area of research remains noteworthy and requires further study and effort to achieve the reconstitution of the entire cycle of gametogenesis and embryo development *in vitro*.

## Introduction

The number of infertile couples has increased from 10 to 15% in the past 10 years. The growing need for assisted reproduction technologies (ART) is a signal for progress in this field. With the development of reproductive medicine, the use of ART is expanding. However, current technologies cannot offer help for patients who lack healthy gametes of their own but yearn for genetically related offspring. Many different pathologies can cause the absence of available gametes. PCOS, ovarian cancer, premature ovarian insufficiency and other ovarian diseases in women and nonobstructive azoospermia (NOA) and chemoradiotherapy of cancer in men are common causes a lack of eggs and sperm. Donor gametes are used in patients for whom ART failed, patients without functional gametes, and homosexual couples who yearn for their genetic offspring. However, this is a passable solution that is not available in many regions and countries. Sperm banking can be a choice for these patients, but it is useless for prepubertal cancer patients, so the cryopreservation of testicular tissues has been applied as a fertility preservation strategy (Goossens et al., 2013; Picton et al., 2015; Gassei and Orwig, 2016). Germ cells obtained from germ cell transplantation or *in vitro* maturation can be used to fertilize oocytes and achieve pregnancy through ART (Brinster and Zimmermann, 1994; Schlatt et al., 2002; Kim, 2006; Kim et al., 2008; Hermann et al., 2012; Saiz and Plusa, 2013). However, it is only helpful for patients who used to have healthy gametes. In addition, for many couples who are bothered with repeated implantation failures and other diseases leading to failed pregnancy, more exploration and specific treatment are needed. However, ethical restrictions are the main impediment for human embryo studies. Protocols for *in vitro* human embryo culture beyond the blastocyst stage remain suboptimal (Deglincerti et al., 2016; Shahbazi et al., 2016). Furthermore, bioethical guidelines prohibit *in vitro* culture of human embryos beyond 14 days post-fertilization or reach the onset of primitive streak (PS) development (Daley et al., 2016; Hyun et al., 2016). Therefore, more research is required in reproductive medicine, and artificial gametes and embryos might be a good platform both in the clinic and for research.

Artificial gametes and embryos can be defined as gametes and embryos generated by manipulation of progenitor cells or somatic cells and stem cells to derive gametes and embryos assemble to their natural state, which provides a new possible therapy for infertility, especially for those people who lack healthy gametes. The ideal goal of artificial gamete production involves gamete formation, fertilization and the birth of offspring, and for embryos, it also requires implantation and development as well as the birth of offspring; these endpoints have been fully achieved. Nevertheless, while this constitutes a barrier, artificial gametes and embryos still represent a promising direction in reproductive medicine. Hopefully, the complete germline will be able to be established *in vitro* in mammalian species, especially in humans.

The generation of artificial gametes and embryos will not only provide therapeutic advantages clinically but also will generate a terrific platform for studying developmental biology. Developmental studies on human germ cells and embryos are mostly based on animal models due to the lack of available human samples. However, gametogenesis and the process of embryo development are species-specific, and the knowledge acquired from animal models cannot be directly translated to humans (Irie et al., 2015; Sugawa et al., 2015). The main reason for interest in artificial gametes and embryos is the possibility of establishing a reproducible method so that ethical issues can be avoided, cellular and molecular events during the developmental process can be well studied, disease models can be established and possible treatment can be developed. The *in vitro* development of human eggs and sperm will pave the way for understanding the complex processes of gametogenesis and for treatment of infertility. In addition, if artificial gametes and embryos can be obtained from patients with diseases, the mechanisms underlying some infertilities could be unraveled, and potential treatment could be explored with this personal disease model. Dominguez et al. (2015) produced pluripotent stem cells from individuals with Turner syndrome, and then the cells were differentiated into germ cell-like cells (GCLCs) and were compared to GCLCs from control individuals. This study revealed that a correct dose of the X chromosome is critical for the maintenance and function of GSCs, which uncovered the mechanism of infertility for Turner syndrome. Patients with inherited genetic disease can obtain healthy progeny without carrying the gene causing the disease if gene editing technology is combined with artificial gamete and embryo technology.

## Gametes From Stem Cells

**FIGURE 1:**
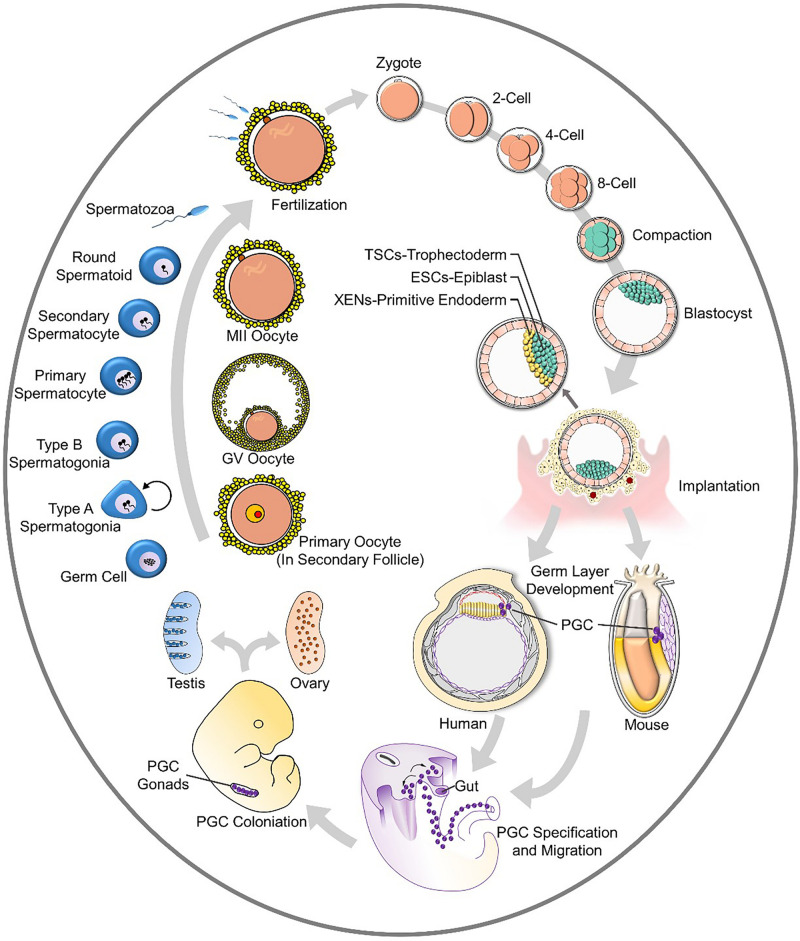
The cycle of gametogenesis and embryo development.

Gametes transmit genetic and epigenetic information through generations (Johnson et al., 2011). Fusion of oocytes and spermatozoa leads to the formation of zygotes, and multistep cleavage gives rise to blastocysts. After implantation, germ layers appear, and the formation and specification of primordial germ cells (PGCs) in the endoderm initiates male and female-specific germ cell development. PGCs develop into germ cells that migrate and colonize before entering into programs of oogenesis or spermatogenesis after puberty. The whole process is generally summarized in Figure 1. This cycling *in vivo* reveals the fundamental stages of gamete formation, and most *in vitro* studies are focused on the establishment of specific cell stages, such as PGCs.

### Reconstitution of PGCs *in vitro*

The key goal of artificial gametes is to produce a functional egg or sperm by reconstituting the process of gametogenesis in culture. Many studies have been focused on the differentiated state of the germ cell lineage.

Primordial germ cells are the precursors of sperm and egg cells, which generate the totipotent state. PGCs arise from the proximal epiblast, which is a region of the early mouse embryo that also contributes to the first blood lineages of the embryonic yolk sac (Lawson and Hage, 1994); PGCs are first observed at the base of the allantois in gastrulating embryos at E7.5 in mice. During gastrulation, a group of mesodermal cells escape from somatic fate; they maintain pluripotency and undergo epigenetic remodeling. In mouse models, PGCs are regulated by BMP4 and BMP8b (Ginsburg et al., 1990; Lawson et al., 1999; Ying et al., 2000), and they upregulate *Prdm1* and *Prdm14* through the mesodermal factor T (Irie et al., 2015; Sugawa et al., 2015). *Tcfap2c1* is downstream of *Prdm1*, which encodes AP2γ and represses somatic gene expression. PGCs are characterized by the expression pluripotent markers such as *Nanog*, *Sox2*, and *Oct4* (Weber et al., 2010) as well as other markers, such as *Fragilis* and *Stella*. PGCs also undergo epigenetic remodeling, including X chromosome activation and a global erasure of DNA methylation patterns. These developments return PGCs to a basal epigenetic state and prepare them or their further differentiation into gametes (Godin et al., 1990; Hackett et al., 2012). PGCs in humans go through a similar pathway of development, but it is not identical. *Prdm14 is* downregulated, and *Sox2* is not expressed.

Primordial germ cells are identified by several key transcription factors (TFs), such as SOX17, TFAP2C and BLIMP1 (also known as PRDM1). These markers are generally used for PGC specification *in vitro* among different species.

Two kinds of stem cells can be used as starting material for generating PGCs: adult stem cells from male and female gonads and pluripotent stem cells, which include embryonic stem cells (ESCs) (Aguilar-Gallardo et al., 2010) and induced pluripotent stem cells (iPSCs) (Takahashi and Yamanaka, 2006; Takahashi et al., 2007; Yu et al., 2007). The pluripotent state of ESCs can be divided into two states: naïve and primed states. The former stage closely resembles the preimplantation epiblast of the blastocyst, while the latter stage resembles the post-implantation epiblast around gastrulation. iPSCs in different species are in different states. Mouse iPSCs mostly resemble the naïve state, and human iPSCs are typically in the primed state. Mouse ESCs are believed to be naïve, while human ESCs are primed (Nichols and Smith, 2009). The two states require different sets of growth factors for their self-renewal and are interchangeable under certain culture conditions. It is also believed that the cellular state that is between naïve and primed ESCs in mice contains the cells that are most easily transformed into PGC-like cells (PGCLCs) (Hayashi et al., 2011).

The derivation of most mouse PGCs from ESCs was accomplished through stepwise differentiation and enables expansion of the cells *in vitro*. Further differentiation is a pathway that stimulates the natural development process *in vivo*. Hayashi et al. reported that they derived mouse PGCLCs from ESCs and iPSCs as well as epiblast-like cells (EpiLCs), a cellular state highly similar to pregastrulating epiblasts but distinct from epiblast stem cells (EpiSCs). Then, further PGC specification was induced with BMP4, BMP8b, LIF, SCF, and EGF. BMP4 plays a vital role in PGC specification, Rolipram and forskolin work in the process of expansion, and RA and BMP2 induce meiosis (Ohta et al., 2017; Miyauchi et al., 2018). The reconstitution of PGCs has also been explored in primates. ESCs and iPSCs from cynomolgus monkeys (*Macaca fascicularis*, referred to as “cy”) are efficiently induced to differentiate into PGCLCs bearing a transcriptome similar to that of early cyPGCs (Sakai et al., 2019). In humans, the specification of PGCLCs has not been achieved. Germline competency and the specification of PGCs are thought to occur in a restricted developmental window during early embryogenesis. Despite the importance of specifying the appropriate number of PGCs for human reproduction, the molecular mechanisms governing PGC formation remain largely unexplored. Studies have shown that the TFAP2C-regulated OCT4 naïve enhancer is involved in human germline formation, and TGFβ and WNT signaling pathways function in PGC formation (Chen et al., 2017, 2018). Improved protocols for producing human PGCLCs are required.

Studies have been undertaken to assess these pathways as they relate to PGCs in more advanced mammals; based on these findings, gametes can be derived.

### Gametes From Adult Stem Cells

Gametes from adult stem cells require expansion and directional differentiation. Adult stem cells have specific pluripotency; these cells can self-renewal and differentiation into limited cell lineages. Adult stem cells rely on the niche *in vivo*. The stem cell niche offers a specific microenvironment containing different metabolic factors, molecular pathway factors, sex steroids, immunologic protection, nutrition and even topology.

In adult males, adult stem cells in testicles are named spermatogonial stem cells (SSCs), and their function has been proven by transplantation (Brinster and Avarbock, 1994; Brinster and Zimmermann, 1994) and lineage tracing of self-renewal and differentiation. SSCs are a population of diploid stem cells that undergo self-renewal, and the complex process of cellular differentiation results in spermatogenesis. In mouse models, the number of SSCs is very limited. It is estimated that SSC only accounts for 0.03% of the whole population of germ cells in testicles (Tagelenbosch and de Rooij, 1993).

The long-term *in vitro* propagation of mouse SSCs (mSSCs) was first published in 2003 (Kanatsu-Shinohara et al., 2003). This protocol was established with several factors secreted by somatic cells in mouse testes, including glial cell line-derived neurotrophic factor (GDNF), leukemia inhibitory factor (LIF), epidermal growth factor (EGF), and basic fibroblast growth factor (bFGF). Other substances were added to this culture medium to build a microenvironment similar to the niche in mouse testes. This work offered inspiration for many studies focusing on SSC *in vitro* propagation, and several kinds of modified media have been applied to different primate cells, including cells from humans (Tesarik et al., 2000; Izadyar et al., 2003; Lim et al., 2010; Kokkinaki and Djourabtchi, 2011; Zheng et al., 2014; Medrano et al., 2016; Gat et al., 2017). However, this medium cannot achieve the expectation of long-term proliferation of SSCs in every type of primate.

The testicular niche consists of two compartments: the interstitial tissue and the seminiferous tubules. Sertoli cells are present inside the tubules and serve as structural support for germ cells. The tubules are surrounded by peritubular myoid cells. Outside the tubules, the insterstitium consists of Leydig cells, macrophages, fibroblasts and blood vessels. The function and fate of SSCs are regulated by the niche, which refers to the microenvironment surrounding SSCs that is mostly constituted by Sertoli cells (SCs). The microenvironment of SSCs differs in different primates; several studies focused on the testicular niche, which is constituted by SCs, the extracellular matrix and the vasculature network (Shinohara et al., 1999; Yoshida et al., 2007; De Rooij, 2009), but subtle differences exist in different species. For example, the number of germ cells that SCs could support is limited and species-specific (Griswold, 1998; Johnson et al., 2008; Schlatt and Ehmcke, 2014; França et al., 2016). SCs are mostly described as being the main supporter for spermatogenesis. This evidence has been supported by *in vitro* experiences. Although spermatogenesis has not been fully achieved *in vitro*, the most successful attempts to this point have been based on co-culture of germ cells with SCs (Griswold, 1998; Nagano et al., 1998; Zanganeh et al., 2013; Rebourcet et al., 2014; Xie et al., 2015; França et al., 2016). Sertoli cells secrete factors that direct germ cell fate. In addition, the metabolic state supported by the Sertoli cell-generated microenvironment also matters. Stem cells, including SSCs, have a tendency to favor the Warburg effect. Therefore, SCs support a glycolytic environment for SSCs (Alves et al., 2014; Oliveira et al., 2015; Meneses et al., 2016; Helsel et al., 2017; Marco and Pedro, 2018). *In vitro* experiments with SSCs from non-human primates and humans cannot reach the standard mSSCs reached, which means establishing a testicular niche *in vitro* may require not only substances secreted by somatic cells but also the spatial structure. In addition, the number of somatic cells could be a large factor in the *in vitro* culture system because of their responsiveness to media additives and because they have shorter proliferation cycles (Gat et al., 2017). SSCs are mostly quiescent. Hereafter, effectively controlling the number of somatic cells in the culture system may trigger the SSCs to proliferate.

For women, the presence of oogonial stem cells (OSCs) in postnatal mammalian ovaries is controversial, as it has long been held that the ovaries contain a fixed number of germ cells throughout a woman’s lifetime (Zuckerman, 1951). However, recent studies have provided evidence of mitotically active OSCs in adult murine and human ovaries (Johnson et al., 2004; Zou et al., 2009; Parte et al., 2011; White et al., 2012). Based on the ability of stem cells, which is a function of the self-renewal and differentiation of the cells, OSCs are believed to be a potentially inexhaustible source of oocytes that can be exploited to achieve fertility in women who are infertile or have an exhausted ovarian reserve, as long as the genetic integrity of the OSCs is maintained (Virant-Klun et al., 2008; Woods and Tilly, 2012; Dunlop et al., 2013; Gheorghisan-Galateanu et al., 2014). Unlike OSCs (expressing nuclear OCT-4B), which are large, another population of small stem cells is believed to exist in the ovary. Very small embryonic-like cells (VSELs, expressing nuclear OCT-4A) are located in the ovary surface epithelium (OSE). There is a postulation that VSELs are the most primitive, pluripotent stem cells in the ovary and that they give rise to committed tissue-specific progenitors, including OSCs, expressing OCT-4 in the cytoplasm as well as other germ cell markers (Virant-Klun et al., 2008; Bhartiya et al., 2014).

Even though the existence of OSCs is in dispute, there have been studies that generated oocytes or oocyte-like cells from OSCs. Investigators found SSEA4+ cells on the human OSE that abundantly expressed markers of primordial and pluripotent germ cells. Tilly and coworkers observed that OSCs isolated by FACS (Virant-Klun et al., 2009, 2013; Stimpfel et al., 2013) differentiated *in vitro* into large mature oocytes that became progressively larger, reaching up to 30–50 μm in diameter; further, they expressed terminal markers such as zona pellucida (ZP) glycoproteins, GDF-9 (growth differentiation factor-9), NOBOX (newborn ovary homeobox), YBX2 (Y-box binding protein 2), SYCP3 (synaptonemal protein complex-3) and molecular modifications typical of a haploid karyotype (White et al., 2012; Zhang et al., 2015).

Similar to SSCs with Sertoli cells, somatic cells also play an important supporting role in oocyte specification. Oocyte-granulosa cell complexes (OGCs) are regarded as the stem cell niche in the female reproductive system. Extracellular matrix signaling activates the differentiation of OSCs in human ovaries, and the function is species-specific. Changes in the tissue microenvironment with age have been postulated to affect ovarian function and failure (Niikura et al., 2009; Massasa et al., 2010). Contact between mouse OSCs (mOSCs) and both type I and type IV collagens activates meiotic differentiation (Stra8 expression) and oogenesis (IVD oocyte formation) through a pathway that involves interaction between the collagens and RGD motif-binding integrin subunits. In comparison, human OSCs (hOSCs) express a pattern of integrin subunits that is different from that of mOSCs, and hOSCs were unresponsive to a collagen-based ECM; however, hOSCs exhibited increased differentiation into IVD oocytes when cultured on laminin (MacDonald et al., 2019).

The pool of stem cells is believed to be balanced. Thus, the expansion of adult stem cells and differentiation into gametes has great potential for clinical use. If SSCs can be expanded and differentiated into spermatids, men who suffer from a lack of efficient gametes, such as those with nonobstructive asthenia (NOA), may be enabled to produce genetically related offspring. If OSCs can be expanded and differentiated into oocytes, this technique can be applied clinically for women who lack oocytes for different reasons, such as PCOS. However, there are still obstacles to overcome before these methods can be applied in the clinic due to the related ethical problems. In addition, whether artificial gametes are healthy enough to produce offspring is still uncertain.

### Gametes From Embryonic Stem Cells

Apart from adult stem cells, ESCs have always been a good platform for studying *in vitro* cell lineage differentiation (Nishikawa et al., 1998; Rathjen et al., 1998). Germ cell differentiation goes through several stages, and studies focus on different stages. The germ cell lineage arises from epiblasts, and they express specific genes that are absent from somatic cells such as *Dazl*, *Piwil2*, *Rnf17*, *Rnh2*, *Tdrd1*, and *Tex14* (Cooke et al., 1996; Seboun et al., 1997; Wang et al., 2001; Moore et al., 2003).

The testes or ovaries of infertile people still contain SSCs or OSCs, and these precursor cells can proliferate and differentiate *in vitro* and may serve a role in assisted reproductive technologies. However, for people lacking adult stem cells, the only remaining option is the transformation of patient-specific somatic cells into pluripotent stem cells, which is then followed by differentiation into genetically related haploid gametes. There are two ways to generate patient-specific PSCs. First, the nucleus of a somatic cell can be transferred into an enucleated oocyte, which is also known as somatic cell nuclear transfer (SCNT) (Tachibana et al., 2013). This oocyte will develop into an embryo at the blastocyst stage, and the ICM can be retrieved. With these cells, a patient-specific ESC line can be generated. Second, somatic cells can be reprogrammed into human induced pluripotent stem cells (hiPSCs) (Takahashi et al., 2007). In general, the iPSC is more commonly used in the initial steps of this process. It is believed that somatic cells constitute an important support system guiding differentiation, but most studies focus on either female or male *in vitro* gametogenesis. Many developed procedures use a stepwise differentiation that is based on the specification of PGCLCs.

The biologically active vitamin A metabolite all-trans RA has two important functions in human spermatogenesis. RA is a key regulator of the transition from undifferentiated spermatogonia into differentiating spermatogonia, and RA plays an important role by promoting the initiation of meiosis in germ cells. In the testis, Sertoli cells synthesize RA from retinol and tightly regulate its distribution to germ cells. All protocols established for *in vitro* spermatogenesis require RA (Hogarth and Griswold, 2010). Male haploid germ cells at the round spermatid stage have been derived from ESCs by spontaneous differentiation *in vitro*. This offers the possibility of investigating germ cell development, epigenetic reprogramming, and germline gene modification. ESC-derived PGCs can differentiate into sperm, as shown by transplantation experiments wherein the form MVH-expressing spermatogenic cells. Injecting ESC-derived PGCs into oocytes can restore the somatic diploid chromosome complement and can enable development into blastocysts (Toyooka et al., 2003; Geijsen et al., 2004). Zhou et al. reported the generation of haploid male gametes from mouse ESCs that can produce viable and fertile offspring, demonstrating functional recapitulation of meiosis *in vitro*. Haploid spermatid-like cells (SLCs) were derived by stepwise differentiation of ESCs. Derived PGCLCs co-cultured with neonatal testicular somatic cells and factors including retinoic acid (RA), BMP4, BMP8a, Activin A, follicle stimulating hormone (FSH), bovine pituitary extract (BPE) and testosterone. This process completely recapitulated meiosis *in vitro*, as shown by the achievement of meiotic hallmarks, and intracytoplasmic injection of SLCs produced euploid and fertile offspring (Zhou et al., 2016). Additionally, due to the interspecies differences in spermatogenesis between rodents and humans, it is inappropriate to simply copy the protocols used in mouse studies and apply them to human cells. Full clarification of the *in vivo* regulators of human spermatogenesis has not yet been obtained. However, regulators such as Activin A, TGFβ, BMP4, GDNF, bFGF, LIF, SCF, EGF, RA, and testicular somatic cell support have been demonstrated to be important mediators of hPSC differentiation toward germ cells of different maturation stages.

For female gametes, appropriate conditions must be available for the three events necessary for *in vitro* reconstitution of oogenesis: the initial phase of meiosis, follicular assembly, and oocyte growth and complete maturation. Many *in vitro* oogenesis attempts have been made in mice using cells from ovaries. Morohaku et al. (2016) used an estrogen inhibitor, ICI182780, to inhibit estrogen-mediated signaling in their organ culture system to prevent the formation of multioocyte follicles (MOFs). Human oogonia can be derived from hiPSCs, which also starts with hPGCLC specification and the co-culture of xenogeneic reconstituted ovaries (xrOvaries) with mouse embryonic ovarian somatic cells. After almost 4 months of culture, cells exhibit characteristics similar to those of human oogonia. Transcriptional information indicates that these cells are undergoing processes such as epigenetic reprogramming, erasing the parental imprints and reactivating the inactive X chromosome (Xi) (Yamashiro et al., 2018). Studies focused on *in vitro* oogenesis and their culturing pathways are summarized in Table 1. We can tell that the culturing system of mouse oogenesis is fully developed, but a similar system cannot be replicated in humans. Obstacles exist, including the complicated events that occur during oogenesis.

**TABLE 1:** Pathways of oogenesis.

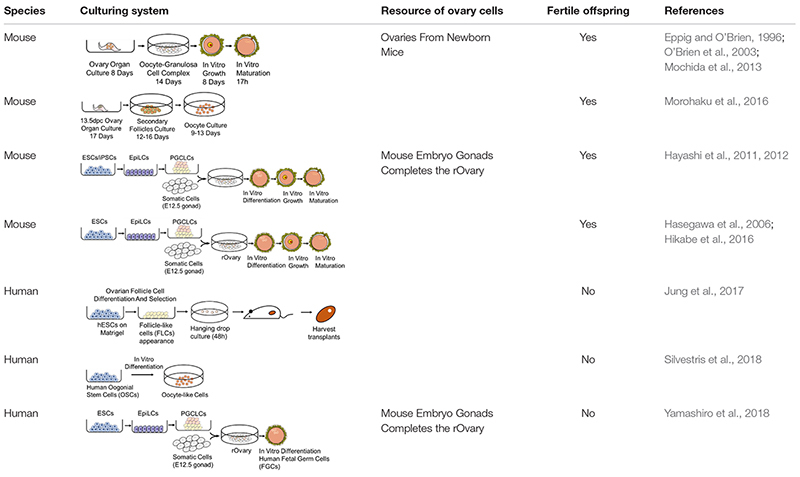

Somatic cells in adult gonads are pivotal for germ cell maintenance and gamete differentiation. Many studies have proven their function and have established protocols for producing artificial gametes. The interaction between somatic cells and germ cells and the 3D structure that somatic cells maintain are crucial for the steady state in adult gonads, which might provide insights into the *in vitro* derivation and expansion of artificial gametes. In addition, these artificial models might explain the relationship between gametes and somatic cells and provide new insights into diseases that cause infertility.

Derivation of gametes from PSCs occurs from a more specific and fixed initial state than derivation of gametes from other sources. However, due to the differences between species, the protocols for mice cannot be directly applied to humans and any other more advanced mammals. Generating fertile offspring with the gametes produced from these procedures is a criterion of success. This is a feasible method for experimental animals but not for humans because of ethical restrictions. Therefore, more suitable protocols and practicable criteria for humans are required.

## Embryos From Stem Cells

The blastocyst (the early mammalian embryo) forms all embryonic and extraembryonic tissues, including the placenta. This structure occurs at the 32-cell stage, which is shortly after the embryo loses its “ball of cells” shapes and forms a cavity. In this structure, the inner cell mass (ICM) will develop into the embryo proper (Nishioka et al., 2009; Stephenson et al., 2010; Hirate et al., 2015; Yu et al., 2016). Cells enveloped around the ICM are trophectoderm (TE) cells, which will later contribute to extraembryonic tissues (fetal placenta and membranes). The ICM further differentiates into the embryonic epiblast and primitive endoderm (PrE) while preparing for implantation (Plusa et al., 2008; Guo et al., 2010; Saiz and Plusa, 2013). Different kinds of stem cells develop from different locations in the blastocyst, as summarized in Figure 1.

Very little is known about the very first steps of human embryonic development due to the small size of the embryo and the limited accessibility in the womb. Several factors may affect implantation, and problems during implantation or shortly after implantation are the main reasons for pregnancy loss at early stages (Norwitz et al., 2001). Generating large numbers of isogenic, accessible embryos of the relevant stage is not possible through *in vivo* or IVF approaches, so an alternative approach would provide a valuable system for studying early pregnancy problems (Pour and Nachman, 2019). Thus, artificial embryos might be a solution for this problem. The goal of artificial embryos is to establish embryo-like structures without any germ cells; some researchers have focused on the differentiation of ESCs to make these structure, while others have found that assembling several types of stem cells might be feasible. The reorganized embryos can be identified through morphological and transcriptomic analysis. The cavity and expression important gene markers are required for successful embryo-like structure establishment. In addition, developmental potency is an important metric.

Many studies have focused on generating blastocyst-like structures by aggregating several kinds of stem cells. Rivron et al. aggregated mouse embryo stem (ES) cells for 24 h and covered these non-adherent aggregates with trophoblast stem (TS) cells to form blastoids that were similar to E3.5 mouse embryos; the formation frequency was low (0.3%). They also proved that cAMP and WNT pathway stimulation can increase TS cell cavitation and blastoid formation and that the ratio of different cell lines is important for the efficiency of blastoid formation (Rivron et al., 2018). There are other studies that have used multiple stem cell types to assemble embryo-like structures. Aggregation of ESCs, TSCs, and/or XENs generated embryo-like structures that recapitulated several key morphogenetic events characteristic of early post-implantation development, including lumenogenesis, epithelialization, and symmetry breaking to specify mesoderm and primordial germ-cell-like cells (Harrison et al., 2017; Sozen et al., 2018; Zhang et al., 2019). Following the identification of EPS cells, which have bidevelopmental potential toward both Em and ExEm lineages (Yang Y. et al., 2017; Yang J. et al., 2017), a method was created that enabled the generation of blastocyst-like structures from mouse EPS cells arose. The embryos they generated resembled blastocysts in morphology and cell lineage allocation and had implantation ability (Li et al., 2019).

Embryonic stem cells are believed to have the property of totipotency. Naïve PSCs are described as cells in the preimplantation BC ICM-like state, and primed PSCs are cells in the post-implantation epiblast-like state. Early attempts to generate embryo-like structures relied on the spontaneous differentiation of ESCs in 3D culture, thereby producing embryoid bodies, which are structurally disorganized cell aggregates (Evans and Kaufman, 1981; Martin, 1981; Doetschman et al., 1985). Kime et al. (2016) converted primed PSCs from mice into a naïve state and then generated BC-like cysts (iBLCs) through sequential culturing (Kime et al., 2019). These iBLCs developed from a totipotent state and expressed important genes but lacked full BC potency. Transcriptome and proteome differences were found between iBLCs and BCs. The implantation and developmental potency of pseudopregnant mice were tested. Cotransferring iBLCs with BCs frequently yielded focal deciduae that were greater in total number than the number of BCs transferred. This might offer new insights for methods to use in cases of difficult embryo transfer in IVF (Mochida et al., 2014). Mouse and human ESCs could establish reorganized embryos resembling a gastrulating embryo when cultured in a soft fibrin matrix and micropatterned condition, respectively (Poh et al., 2014; Warmflash et al., 2014). Moreover, pulsing mouse ESC aggregates in a shaking 3D culture undergoing differentiation with a WNT-b-catenin pathway activator gave rise to elongated gastruloids (Beccari et al., 2018).

The construction of embryo-like structures *in vitro* can offer a model for studying fundamental biological questions in both preimplantation and early post-implantation mammalian embryogenesis and can enable the modeling of diseases related to early pregnancy, the performing of high-throughput pharmacological and toxicological screens, and possibly the bioengineering of mammalian embryos. The development of early mammalian embryos is plastic and is regulated by several evolutionarily conserved developmental processes that can be recapitulated *in vitro*. Artificial embryos are desired not only in mice but also in other mammalian species, including humans. However, the derived reorganized embryos exhibit features of different embryonic stages, but they are not equivalent to totipotent blastomeres. A deeper understanding of these differences is required to build a better *in vitro* environment for natural embryos. In addition, to date, no artificial embryos derived from the organization of stem cells develop normally, and fertile offspring have not been reported. This indicates that artificial and reorganized embryos remain a substantial challenge in the field, and more investment and research are required.

## RNA-seq Offers Insights Into Artificial Germ Cell and Embryo Development

Since RNA sequencing was developed a decade ago (Emrich et al., 2007; Lister et al., 2008), it has become a ubiquitous tool in molecular biology that is shaping nearly every aspect of our understanding of genomic function. Beyond bulk RNA analysis, single-cell analysis and spatially resolved RNA-seq offer deeper information for the answering of biologic questions (Tang et al., 2009; Montoro et al., 2018). Based on single-cell transcriptional profiling and characterization, cells can be utilized not only to determine an accurate picture of cellular stages but also for bulk studies of embryogenesis, maturation, and pathological conditions or regeneration. Single-cell RNA sequencing offers insights into building an *in vitro* culturing system for gametes and embryos. The identification of different stages of gamete differentiation and embryo development is crucial for artificial gametes and embryos. As we discussed above, the number of adult stem cells is very small in adult gonads, and the cells are difficult to evaluate and isolate. Single-cell analysis introduces the possibility of identifying this small cluster of cells.

A single-cell RNA sequencing (scRNA-seq) analysis of 2,854 human testicular cells provides insight into the possible development process of human spermatogenesis, and several pivotal signaling pathways, including the BMP and FGF pathways, have been found to be involved in human SSC self-renewal (von Kopylow et al., 2016; Wang et al., 2018). ScRNA-seq of various stages of oocyte maturation offers transcriptional proteomic and metabolic information of the stages, and comparison of oocytes in healthy women and patients with PCOS reveals that they are dysfunctional in meiotic maturation, gap junctions, hormone responses, DNA damage, and in the factors they secrete in the early GV phase. Thus, meiosis of oocytes at the GV stage is delayed by malfunctioning genes, which may also hinder fertilization and other processes (Liu et al., 2016; Virant-Klun et al., 2016). Comparison of blastocyst-like cysts and blastoids has mainly been performed at the transcriptional level. Markers for the three lineages of cells, trophectoderm, polarization, X inactivation, development potential and implantation, were all tested and compared between blastocysts and reorganized embryos. Instead of testing several genes in artificial gametes and embryos, scRNA-seq offers a complete picture of how similar they are to natural gametes and embryos (Li et al., 2019).

RNA-seq analysis offers researchers a powerful tool for gathering information to help portray the detailed environment and state of natural gametogenesis and embryo development. Differences between species can also be revealed by such methods, and modified procedures can be established based on animal models.

## The Possible Uses of Artificial Gametes and Embryos

Artificial gametes and embryos have scientific uses. Due to ethical restrictions, there are areas where little is known in human embryonic development. The processes of gametogenesis and embryonic development could be better understood. With isogenic gametes and embryos, further culture of human embryos might be achieved, and the processes of early embryo development might be described. Disease models could be established. iPSC technology has brought hope for disease model establishment *in vitro* and treatment for some diseases (Takahashi and Yamanaka, 2006; Takahashi et al., 2007; Han et al., 2016; Paquet et al., 2016; Qian et al., 2016), and we believe that artificial gametes and embryos have the same potential. The mechanisms behind causes of infertility, such as PCOS, endometriosis and gamete developmental disorders, might be explored with the construction of a patient disease model *in vitro*. Treatment could be well developed, and it might even be customized. Embryo resorption represents the failure of implantation (Cossée et al., 2000).

Apart from the research purpose, artificial gametes and embryos are expected to have clinical use; they might represent another available treatment for infertility. Instead of donated sperm or eggs, artificial gametes and embryos could bring hope for genetically related offspring. In addition, patients who lost their fertility because of cancer treatment, including pediatric cancer patients, might regain fertility. These tools also offers another choice of fertility preservation. The safety of artificial gametes and embryos is of special concern. Although fertile offspring can be derived in several rodent studies, these protocols cannot guarantee success in more advanced mammalian species, including humans.

Artificial embryos will become a powerful research platform for early embryo development, especially human embryonic development.

## Discussion

Genetic and epigenetic information is transmitted through the cycle of gametogenesis and embryo development. The reconstitution of the entire cycle of gametogenesis and embryo development *in vitro* is the goal behind the generation of artificial gametes and embryos. Gametogenesis can be initiated from ESCs or adult stem cells. The number of adult stem cells is very limited in both male and female gonads; therefore, expansion of adult stem cells is a critical step before gametogenesis. For ESCs, the limited number of cells is no longer a concern, but it always takes multiple steps to accomplish differentiation. Whether important information has been fully maintained is uncertain, and whether deriving artificial gametes directly from the PSC stage is feasible and preferable is also unknown. The experience derived from mouse models can be inspiring for humans but cannot be translated directly. More experience is required on advanced mammalian species, such as nonhuman primates and humans. The significance of the stem cell niche in the testis and ovary is realized but not fully understood in humans; thus, reconstitution cannot be fully implemented. The field of artificial embryos has just started, and additional work is required.

The development of RNA sequencing started a revolution in biology and medicine, and with this powerful tool, researchers can elucidate the complex relationships between subtypes of cells in the context of sequential cell fate determination in gametogenesis and can pave the way for identifying molecules involved in embryo development. We believe that further investigation of reproduction-related disorders can be performed with RNA sequencing tools. The future of artificial gametes and embryos is profound and lasting. They have both possible scientific and clinical uses, and they might represent a powerful tool for reproductive medicine because they offer potential treatments for infertility and a model of embryo development without the concerns of ethical problems. Artificial gametes and embryos might also be a remarkable tool for rare disease model establishment. In summary, more investment and research are needed in this area.

## Author Contributions

P-YZ and YF designed the study and drafted the manuscript. TT and YY designed the study and supervised the project. All authors contributed to the discussion and approved the final version.

## Conflict of Interest

The authors declare that the research was conducted in the absence of any commercial or financial relationships that could be construed as a potential conflict of interest.
